# Cerebral venous thrombosis in elderly patients

**DOI:** 10.1111/ene.16504

**Published:** 2024-10-04

**Authors:** Victor Garcia, Louise Bicart‐Sée, Isabelle Crassard, Nicolas Legris, Mathieu Zuber, Fernando Pico, Céline Guidoux, Michael Obadia, Naouel Boulenoir, Didier Smadja, Mikael Mazighi, Cecile Lavenu‐Bombled, Elodie Baudry, Bertrand Lapergue, Guillaume Turc, Philippe Tuppin, Christian Denier

**Affiliations:** ^1^ Stroke Units and Department of Neurology of Hôpital Bicêtre, Le Kremlin Bicêtre, Assistance Publique–Hôpitaux de Paris Paris Saclay University Paris France; ^2^ Hôpital Lariboisière, Assistance Publique‐Hôpitaux de Paris, University of Paris City, INSERM 1144 Paris France; ^3^ Hôpital Saint‐Joseph Paris France; ^4^ Hôpital André Mignot Versailles France; ^5^ Hôpital Bichat, Assistance Publique‐Hôpitaux de Paris Paris France; ^6^ Hôpital Fondation Rothschild Paris France; ^7^ Hôpital Sud Francilien Paris France; ^8^ Department of Hematology Hôpital Bicêtre, Assistance Publique‐Hôpitaux de Paris Paris France; ^9^ Department of Geriatry Hôpital Bicêtre, Assistance Publique‐Hôpitaux de Paris Paris France; ^10^ Hôpital Foch Suresnes France; ^11^ Hôpital Sainte‐Anne Paris France; ^12^ French National Health Insurance (CNAM) Paris France

**Keywords:** cerebral venous thrombosis, cohort, elderly, etiology, incidence, prognosis, risk factors, thrombophlebitis

## Abstract

**Background and purpose:**

We aimed to report the characteristics of cerebral venous thrombosis (CVT) in elderly people (aged ≥65 years).

**Methods:**

This multicenter retrospective cohort included elderly patients hospitalized for a first CVT in nine Paris–Ile‐de‐France hospitals between 2011 and 2021. The estimated incidence was compared to CVT recorded by the French health insurance data system. Lariboisière Hospital's CVT registry allowed comparisons of our elderly cohort with individuals younger than 65 years.

**Results:**

One hundred fourteen patients were included in this study (mean age = 74.2 years, range = 65–93, 61% female). The CVT annual incidence in Ile‐de‐France was 5.9–7.1 per million elderly individuals versus 8.5 per million nationwide. Headaches and focal deficits were the most common initial clinical features (50% and 51%, respectively), followed by seizures and confusion (40% and 27%). Treatment included anticoagulation (93%) and, rarely, endovascular procedure (2%) or craniectomy (1%). Compared with adult patients aged <65 years (younger adults), elderly patients presented fewer headaches (50% vs. 96%, *p* < 0.01) and intracranial hypertension (7% vs. 22%, *p* < 0.01) but more seizures and focal deficits (40% vs. 27% and 51% vs. 38%, respectively, *p* < 0.01). Underlying cancer, hemopathy, and locoregional infections were more frequent in elderly patients than among younger adults (*p* < 0.01). The prognosis of patients from our elderly cohort was poorer than that of younger adults; 8% died in the acute phase, and 73% had a favorable outcome at 1 year (vs. 1.7% and 87%, respectively, *p* < 0.01).

**Conclusions:**

CVT in elderly patients has a specific clinical presentation, epidemiology, and risk factors such as cancer or hemopathy, justifying specialized management.

## INTRODUCTION

Cerebral venous thrombosis (CVT) is a rare but potentially severe condition, especially following diagnostic delay. It is less frequent than other types of stroke (0.5%–1% of all strokes) and can be more challenging to diagnose. CVT predominates in young adults, especially women, and can also involve young children, including neonates [1, 2]. The sex ratio differs by age, with an initial male predominance in neonates and children, and a female predominance among adolescents and young and middle‐aged adults [1, 2]. Its incidence comprises between 13 and 20 cases per million inhabitants per year [3, 4]. We recently reported that the CVT incidence in adolescents was 3.7–3.8 per million adolescents per year [2]; however, it remains unknown in the population aged ≥65 years. CVT‐related mortality and morbidity have decreased in recent years [5] thanks to increasingly accessible diagnostic methods and to a better understanding of its clinical presentation among neurologists, emergency physicians, and general practitioners. However, CVT is a time‐sensitive condition, associated with a risk of intracranial hypertension, edema, hemorrhage, and mortality. In this regard, elderly people are particularly at risk, having increased frailty and potentially different and more misleading clinical manifestations. In addition, there are possibly numerous and specific etiologies. Therefore, it is of utmost importance to detect the underlying mechanism or pathology that may have caused or favored CVT and adapt treatment. Currently, the literature on CVT among patients aged ≥65 years is scarce, with only one study focusing on this population (*n* = 51 patients aged >65 years with *n* = 21 aged >75 years) [6] and a recent retrospective study dedicated to the comparison of CVT in young adults with CVT in adults aged >55 years [7].

The purpose of the study is to describe the incidence of CVT in patients aged ≥65 years and their clinical, etiological, and prognostic characteristics, and to compare this group with younger adult patients with CVT. We aimed to provide useful data to clinicians managing elderly patients presenting with CVT.

## METHODS

### Study design and patients

We conducted a retrospective multicenter cohort study in the Paris–Ile‐de‐France region, which represents a population of nearly 10 million inhabitants, by studying the databases of nine French university hospitals. The participant centers were the Bicêtre, Lariboisière, Saint‐Joseph, Mignot, Bichat, Sud Francilien, Rothschild, Foch, and Sainte‐Anne hospitals. We included every patient with a first‐ever diagnosis of symptomatic CVT by magnetic resonance imaging (MRI) or computed tomography (CT) scan, who were aged ≥65 years and had been hospitalized in a stroke unit or a neurological department between January 2011 and November 2021. Patients were screened via a search of the diagnosis according to the International Classification of Diseases, 10th Revision (ICD‐10; thrombophlebitis, cerebral infarction due to CVT) in the participating hospitals (Programme de médicalisation des systèmes d'information [PMSI], the French national hospital discharge database), providing a comprehensive list of target patients. CVT definition comprised evidence of thrombosis on venous sequences (time of flight and T1 gadolinium) on MRI or on CT venography.

### Standard protocol approvals, registrations, patient consent, and ethics

This study was approved by the local institutional review board/independent ethics committee (IRB/IEC; CPP‐VII). As this is a retrospective observational study with anonymized data, without any additional therapy or monitoring procedure, the IRB/IEC did not identify ethical issues and declared that, in accordance with French legislation, formal approval was not required.

### Data collection

We reviewed medical records of all identified patients using a standardized approach to collect several data types: demographic data, preexisting medical conditions, clinical presentation, time between symptom onset and diagnosis, treatment modalities, type and results of the diagnostic and etiological workup (including cerebral imaging to determine involved sinus and parenchymal lesions, usual blood tests, cerebrospinal fluid [CSF] analysis, thrombophilia investigation, and any more specialized analyses), retained associated conditions/risk factors, and clinical outcome (at 3 months and 1 year).

### Clinical features in the acute phase

We collected the proportion of patients with the following modes of presentation of CVT: confusion, headache, isolated headache, isolated intracranial hypertension (headache with CSF pressure ≥ 25 cm H_2_O, or papilledema), focal deficit, seizures, and coma. Symptom onset was defined as acute (<48 h), subacute (<1 month), or chronic (>1 month). Time before medical visit, number of visits before diagnosis, time before diagnosis (defined as time between symptom onset and diagnosis confirmed by imaging), and diagnosis delay (time between first medical visit and diagnosis) were noted when available.

### Etiological investigations

We gathered all information regarding associated medical conditions that could be potential underlying risk factors (noting that causality cannot be proven). The medical conditions that were considered relevant were the generally admitted risk factors for CVT, such as active cancer, hemopathy (acute or chronic), *JAK2* mutations, nonacquired thrombophilias (antithrombin III, protein S or protein C deficiency, factor V and II mutation), antiphospholipid syndrome (APS; confirmed with 3‐month hindsight), local or systemic infection, inflammatory diseases, and/or hormonal exposure.

### Outcome

We collected the following information: in‐hospital mortality, hemorrhagic complications, and deaths occurring during the follow‐up, ability to return home after hospitalization, frequency of motor and cognitive impairment, and clinical outcome at 3 months and 1 year after CVT, which was assessed by the modified Rankin Scale (mRS). A good functional status was defined as an mRS of 0–1.

### Incidence

In France, the national census regularly provides detailed population data according to age/region. We used these data to estimate the population of patients aged ≥65 years in the Paris–Ile‐de‐France area covered by participating centers during the study period. Annual incidence of CVT in people aged ≥65 years in the aforementioned area was then estimated from the number of patients included in our study compared with the population data.

### Data validation and comparison with national data from the hospital discharge database

Considering the retrospective nature of our study and that some hospitals of the Ile‐de‐France region did not participate, we decided to confirm the reliable identification of all potential cases despite the retrospective nature of our study. Thus, we compared the number and age distribution of first‐ever CVT observed in our study to those in the total Ile‐de‐France and overall French population for the same age category over the corresponding period of time (2011–2021) using data extracted from the French national discharge database (ICD‐10 coding) [8]. In France, the national health insurance information system contains individual, anonymized data concerning the beneficiaries of the various national health insurance schemes for the entire French population [8]. Using these databases, we were able to compare the age‐specific distribution in our study population with that of the total French and Ile‐de‐France region population.

### Subgroup comparisons

Despite modest sample size, we made exploratory analysis and compared several characteristics according to sex (female/male) or age (dividing our cohort into two groups of people aged ≤75 years or >75 years).

### Comparison with the prospective Lariboisière register of adult patients with CVT younger than 65 years

Some characteristics of our cohort (≥65 years old with CVT) were compared with the prospective Lariboisière CVT register of 478 adults aged <65 years, recruited from 1998 to 2020. The Lariboisière register gathered clinical, etiological, management, and prognostic data [2].

### Statistics

Statistical comparisons were made for different features. First, we made comparisons among patients aged ≥65 years between male and female patients and comparisons between the youngest and oldest patients (65–74 years vs. ≥75 years). Second, we also compared patients aged ≥65 years and adult patients aged <65 years from the Lariboisière register. Descriptive data are reported associated with the percentage and either mean and SD or median and interquartile range (IQR). Distributions were compared by use of a *χ* ^2^ test when the number of patients per both groups was ≥5 and with the Fisher exact test when the number of patients in at least one group was <5. The Mann–Whitney test was used to analyze mean age among patients aged ≥65 years owing to the nonparametric distribution of ages. We performed a univariate analysis with a test of logistic regression to identify patient characteristics associated with a good prognosis (mRS = 0–1). Results are given with odds ratios (ORs) and 95% confidence intervals (CIs).

## RESULTS

### Population characteristics and demographic data

**FIGURE 1:**
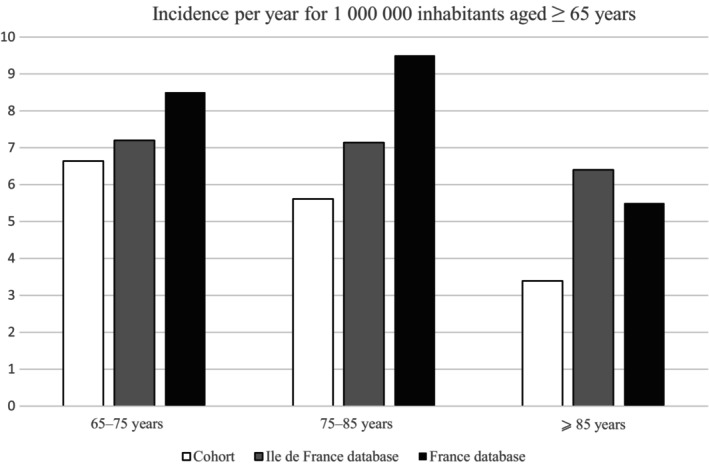
Annual incidence of cerebral venous thrombosis (CVT) according to age in our cohort, the Ile‐de‐France region, and France overall. Estimated incidence of CVT in patients aged ≥65 years was calculated from the symptomatic CVT cases of our cohort (white), and using data extracted from the French national discharge database (International Classification of Diseases, 10th Revision coding) in the Ile‐de‐France region (gray) and France overall (black).

One hundred fourteen symptomatic patients aged ≥65 years with a first CVT presenting between January 2011 and November 2021 were identified at the participating hospitals (4–29 patients per center). Most patients were female (61%). The median age at CVT occurrence was 72.6 years (IQR = 67.8–79.0), and the oldest patient was 93 year old (Table 1). The incidence of first‐ever symptomatic CVT in patients was 5.9 per million inhabitants aged ≥65 years per year in our cohort according to our analysis of medical records. According to data from the French national discharge database, yearly incidence of first‐ever symptomatic CVT was 7.1 per million inhabitants aged ≥65 years in the Ile‐de‐France population and 8.5 per million in France overall. Figure 1 shows the incidences stratified by age group. In our cohort, as in the Ile‐de‐France national health insurance database, CVT incidence decreased with age.

**TABLE 1:** Whole cohort of symptomatic CVT patients aged ≥65 years and comparison of two age subgroups (<75 years old and ≥75 years old).

Characteristic	All, *N* = 114	Age < 75 years, *n* = 71	Age ≥ 75 years, *n* = 43	*p*
Female, *n* (%)	69/114 (61)	38/71 (54)	31/43 (72)	**0.049**
Previous history, *n* (%)				
Venous thrombosis	9 (9)	5 (7)	4 (5)	NS
Inflammatory disease	4 (4)	4 (6)	0 (0)	
Cancer in remission^a^	12 (11)	7 (10)	5 (12)	
Symptom onset, *n* (%)				
Acute	51/110 (46)	26/67 (39)	25/43 (58)	NS
Subacute	46/110 (42)	31/67 (46)	15/43 (35)	
Chronic	13/110 (12)	10/67 (15)	3/43 (7)	
Median time, days (IQR)				
From symptom onset to first consult	5 (3–21), *n* = 98	7 (3–21), *n* = 60	2 (0–9), *n* = 38	<0.01
From first consult to diagnosis	0 (0–1), *n* = 93	0 (0–1), *n* = 55	0 (0–1), *n* = 38	NS
Clinical presentation, *n* (%)				
Confusion	31/114 (27)	19/71 (27)	12/43 (28)	NS
Isolated headache	21/114 (18)	17/70 (24)	4/44 (9)	**0.04**
Headache	57/114 (50)	41/71 (58)	16/43 (37)	**0.03**
Isolated intracranial hypertension	8/111 (7)	7/68 (10)	1/43 (2)	NS
Seizure	46/114 (40)	26/71 (37)	20/43 (17)	NS
Focal deficit	58/113 (51)	29/70 (41)	29/43 (67)	**<0.01**
Papilledema	7/40 (18)	6/27 (22)	1/13 (8)	NS
Coma [Glasgow score ≤ 8]	11/114 (10)	6/71 (8)	5/43 (12)	NS
CVT diagnosis based on, *n* (%)				
MRI	64/113 (57)	42/70 (60)	22/43 (51)	NS
TDM	49/113 (43)	28/70 (40)	21/43 (49)	
Thrombosis location, *n* (%)				
Lateral sinus	79/114 (69)	54/71 (76)	25/43 (58)	**< 0.01**
Superior sagittal sinus	49/114 (43)	31/71 (44)	18/43 (42)	NS
Cortical vein	32/114 (28)	17/71 (24)	15/43 (35)	NS
Deep/straight vein	16/114 (14)	9/71 (13)	7/43 (16)	NS
Cavernous sinus	4/114 (3)	2/71 (3)	2/43 (5)	NS
Multiple locations	52/114 (46)	33/71 (46)	19/43 (44)	NS
Brain injury, *n* (%)				
Venous infarction	28/113 (25)	15/70 (21)	13/43 (30)	NS
Cerebral hemorrhage	34/113 (30)	16/70 (23)	18/43 (42)	**0.03**
Subarachnoid hemorrhage	18/113 (16)	5/70 (7)	13/43 (30)	**<0.01**
Dural arteriovenous fistula	6/114 (5)	5/71 (7)	1/43 (2)	NS
Associated conditions, *n* (%)				
Congenital thrombophilia^b^	9/72 (13)	6/51 (12)	3/21 (14)	NS
Hemopathy^c^	8/109 (7)	5/69 (7)	3/40 (8)	NS
Cancer [active]^d^	15/109 (14)	9/69 (13)	6/40 (15)	NS
Local infection [face or intracranial]^e^	9/109 (8)	6/69 (9)	3/40 (8)	NS
Nonlocal infection	5/109 (5)	1/69 (1)	4/40 (10)	NS
Antiphospholipid syndrome	5/71 (7)	4/51 (8)	1/20 (5)	NS
*JAK2* mutation	4/18 (22)	3/10 (30)	1/8 (13)	NS
Other investigations, *n* (%)				
Cancer searched for (by CT or PET‐CT)	(69)	(71)	(65)	NS
Anemia [hemoglobin<10 g/dL]	(6)	(9)	(0)	NS
Thrombopenia [platelets<150,000/mm^3^]	(9)	(9)	(8)	NS
Thrombocytosis [platelets>450,000/mm^3^]	(6)	(3)	(11)	NS
Elevated CRP [if>10]	(41)	(36)	(49)	NS
Lumbar puncture done	35/113 (31)	25/70 (36)	10/43 (23)	NS
Pleocytosis [>5/mm^3^]	4 (12)	4/25 (16)	0 (0)	NS
ICH	3/10 (30)	3/8 (38)	0/2 (0)	NS
Anticoagulation, *n* (%)	105/113 (93)	68/71 (96)	37/42 (88)	NS
Anticoagulation duration				
Mean duration if temporary, months (SD)	10.8 (13.6)	10.9 (10.1)	10.6 (19.6)	NS
For life, *n* (%)	24/65 (37)	19/45 (42)	5/20 (25)	
Long‐term anticoagulation treatment, *n* (%)				
DOAC	25/100 (25)	11/66 (16)	14/34 (41)	**<0.01**
Vitamin K antagonists	53/100 (53)	42/66 (64)	11/34 (32)	
Antiepileptic therapy, *n* (%)	42/114 (37)	23/71 (32)	19/43 (44)	NS
Thrombolysis/thrombectomy, *n* (%)	2/114 (2)	2/71 (3)	0/43 (0)	
Craniectomy, *n* (%)	1/114 (1)	1/71 (1)	0/43 (0)	
Hemorrhagic complication, *n* (%)	8/110 (7)	6/68 (9)	2/42 (5)	
Death in the acute phase, *n* (%)	9/113 (8)	1/70 (1)	8/43 (19)	**<0.01**
Able to return home after hospitalization, *n* (%)	76/100 (76)	54/63 (86)	22/37 (59)	**<0.01**
Motor impairment, *n* (%)	13/83 (16)	4/53 (8)	9/30 (30)	**0.01**
Cognition impairment, *n* (%)	17/82 (21)	8/53 (15)	9/29 (31)	NS
mRS at 3 months, *n* (%)				
0	41/69 (60)	31/45 (69)	10/24 (42)	**<0.01**
1	7/69 (10)	5/45 (11)	2/24 (8)	
2	3/69 (4)	2/45 (11)	1/24 (4)	
3	4/69 (6)	2/45 (11)	2/24 (8)	
4	4/69 (6)	3/45 (7)	1/24 (4)	
5	1/69 (1)	1/45 (2)	0/24 (0)	
6	1/69 (1)	1/45 (2)	8/24 (33)	
mRS at 1 year, *n* (%)				
0	41/63 (65)	32/43 (74)	9/20 (45)	**<0.01**
1	5/63 (8)	4/43 (9)	1/20 (5)	
2	2 /63 (3)	2/43 (5)	0/20 (0)	
3	3/63 (5)	1/43 (2)	2/20 (10)	
4	3/63 (5)	3/43 (7)	0/20 (0)	
5	0/63 (0)	0/43 (0)	0/20 (0)	
6	9/63 (14)	1/43 (2)	8/20 (40)	

Abbreviations: CRP, C‐reactive protein; CT, computed tomography; CVT, cerebral venous thrombosis; DOAC, direct oral anticoagulants; ICH, intracranial hypertension; IQR, interquartile range; MRI, magnetic resonance imaging; mRS, modified Rankin Scale; NS, not significant; PET, positron emission tomography; TDM, therapeutic drug monitoring.

^a^ Gastrointestinal, melanoma, prostate, ear/nose/throat area, or breast cancer.

^b^ Factor V Leiden or factor II mutations, protein C and S or antithrombin deficiency.

^c^ Myeloma and chronic myeloproliferative neoplasms.

^d^ Lung, breast, prostate, gastrointestinal, thyroid, ovary, and endometrium cancer.

^e^ Bacterial meningitis, sinusitis, mastoiditis, subdural empyema, parotiditis.

### Presentation and diagnosis

Characteristics of patients aged ≥65 years are presented in Table 1. Symptom onset was predominantly acute or subacute (46% and 42%, respectively) and rarely chronic (12%). The median time from symptom onset to diagnosis was 5 days, and diagnosis was made in the first 48 h in most cases (64%). The two most common clinical features were headaches (57/114, 50%) and focal deficits (58/113, 51%). More than half of patients experiencing headaches had another associated clinical feature (32/57, 56%). Seizures and confusion were also common (46/114, 40% and 31/114, 27%, respectively). Among patients who presented with seizures, it was the sole manifestation for 30% of them. Funduscopic examination was performed for only 40 patients (35%), and papilledema was identified in seven (18%).

The thrombus location was a lateral sinus in 69% of cases, the superior sagittal sinus in 43% of cases, and in multiple locations in 46% of cases. Cortical vein thrombosis was observed in 32 patients (28% of cases), including six patients without involvement of either dural sinuses or the deep venous system. Fifty‐two patients of our cohort (46%) had parenchymal damage, with venous infarction (25%), cerebral hemorrhage (30%), or both venous infarction and cerebral hemorrhage (9%).

### Associated risk factors

Hemopathies and cancers were the most common CVT‐associated conditions. In cases where there was no history of known cancer, it was searched for by systematic screening by CT or positron emission tomography scan in 73 of 106 patients (69%). Overall, there were 15 cases (14%) of active cancer and eight cases (7%) of hemopathy (acute and chronic). A *JAK2* mutation was discovered four of the 18 times it was searched for (22%), plus one patient for whom the mutation was already known, as he had Vaquez polyglobulia. Only three of the five patients with a *JAK2* mutation had thrombocytosis or polyglobulia. There were four cases of inflammatory rheumatological disease, three cases of rheumatoid polyarthritis, and one case of spondylarthritis. Lumbar puncture was performed for 35 patients in our cohort; there were only two cases of bacterial meningitis. The ear, nose, and throat (ENT) area infections (8/114, 7%) were mainly sinusitis (5/8, 63%); there were two cases of mastoiditis and one parotiditis with face infection. There were no cases associated with SARS‐Cov‐2 infections or COVID‐19 vaccines. Congenital thrombophilia and antiphospholipid syndrome were searched for in most patients (63%). We discovered nine patients in our cohort who had congenital thrombophilia; there were eight heterozygous mutations of the prothrombin gene and one heterozygous factor V gene mutation, two patients had protein C deficiency, and one had antithrombin deficiency. A diagnosis of APS was made in five cases. A definite associated condition (any of the conditions mentioned above) was found in 45 patients (39%). Only one female patient was receiving hormone replacement therapy (HRT) before CVT diagnosis.

### Therapeutic management, outcome, and prognosis

Anticoagulant therapy was administered to 93% of patients. One patient was treated with thrombolysis, thrombectomy, and craniectomy, and another patient was treated with thrombectomy alone. Nine patients of the cohort (8%) had a fatal outcome; all the deaths occurred in the acute phase or during the first 3 months following the CVT. Most patients were able to return home at the end of hospitalization (76%), and most had a good prognosis at 3 months, namely, an mRS of 0–1 (70%). After univariate analysis, seven factors were negatively associated with this good prognosis (OR < 1): age (OR = 0.90, 95% CI = 0.83–0.97, *p* < 0.01), confusion (OR = 0.27, 95% CI = 0.08–0.84, *p* = 0.02), focal deficit (OR = 0.21, 95% CI = 0.07–0.63, *p* = 0.01), coma (Glasgow score ≤ 8; OR = 0.05, 95% CI = 0.01–0.48, *p* = 0.01), deep/straight vein thrombosis (OR = 0.12, 95% CI = 0.03–0.46, *p* < 0.01), venous infarction (OR = 0.27, 95% CI = 0.09–0.86, *p* = 0.03), and cerebral hemorrhage (OR = 0.13, 95% CI = 0.04–0.46, *p* < 0.01; Table 2).

**TABLE 2:** Predictive factors of good prognosis (modified Rankin Scale = 0–1) at 3 months following univariate analysis.

Variable	OR	95% CI	*p*
Age	0.90	0.83–0.97	**<0.01**
Sex	0.95	0.34–2.69	0.93
Time from symptom onset to first consult	1.04	1.00–1.10	0.07
Headache	1.27	0.45–3.57	0.65
Intracranial hypertension	5.81	0.70–48.43	0.10
Confusion	0.27	0.08–0.84	**0.02**
Seizure	0.37	0.13–1.08	0.07
Focal deficit	0.21	0.07–0.63	**0.01**
Coma (Glasgow score < 8)	0.05	0.01–0.48	**0.01**
Fever	0.49	0.13–1.85	0.29
Lateral sinus thrombosis	1.45	0.45–4.71	0.53
Deep/straight vein thrombosis	0.12	0.03–0.46	**<0.01**
Superior sagittal sinus thrombosis	0.55	0.19–1.54	0.25
Cortical vein thrombosis	0.93	0.30–2.91	0.90
Venous infarction	0.27	0.09–0.86	**0.03**
Cerebral hemorrhage	0.13	0.04–0.46	**<0.01**
Subarachnoid hemorrhage	0.56	0.15–2.03	0.38

Abbreviations: CI = confidence interval; OR = odds ratio.

### Comparison between male and female patients within our cohort

Male patients were significantly younger than female patients at the time of CVT occurrence (72.5 vs. 75.3, *p* = 0.04), and the proportion of female patients significantly increased with age (54% of patients <75 years old vs. 72% of patients aged ≥75 years old). There was no difference in the clinical pattern of symptoms, either for timing from onset to diagnosis, thrombus location, underlying pathology, or outcome between male and female patients (data not shown); we only found significantly more venous infarction among women (29% vs. 12%, *p* = 0.02).

### Comparison between two age subgroups within our cohort

We subsequently compared patient characteristics between two age subgroups within our group: patients aged 65–74 years (*n* = 71) and patients aged ≥75 years (*n* = 43; Table 1). Symptom onset was similarly acute, subacute, and chronic in these two groups. The median time from symptom onset to diagnosis was shorter for patients of the older subgroup (2 days, IQR = 0–9 vs. 7 days, IQR = 3–21). The proportion of patients experiencing headaches as an isolated symptom was significantly lower for the older subgroup than the younger (9% vs. 24%, respectively, *p* = 0.04), and the proportion experiencing headaches as one of several symptoms was also significantly lower for the older subgroup (37% vs. 58%, respectively, *p* = 0.03); they also had more focal deficits (67% vs. 41%, *p* < 0.01; Table 1). Hemorrhagic complications were significantly more frequent among the older than younger subgroup, 42% versus 23% for cerebral hemorrhage (*p* = 0.03) and 30% versus 7% for subarachnoid hemorrhage (*p* < 0.01). There was no difference in associated conditions or underlying pathology. The prognosis was poorer for the older subgroup than the younger. Eight of the nine fatal outcomes in the acute phase concerned patients aged ≥75 years, making the death rate significantly higher in the older group (*p* < 0.01). Within our cohort, younger patients had a better outcome at 3‐month and 1‐year follow‐up. At the end of hospitalization, the proportion of patients in the younger subgroup having a motor impairment was lower (8% vs. 30%, *p* = 0.01) and the proportion being able to return home was higher (86% vs. 59%, *p* < 0.01).

### Comparison of our cohort with symptomatic CVT in adults younger than 65 years from the Lariboisière register

**FIGURE 2:**
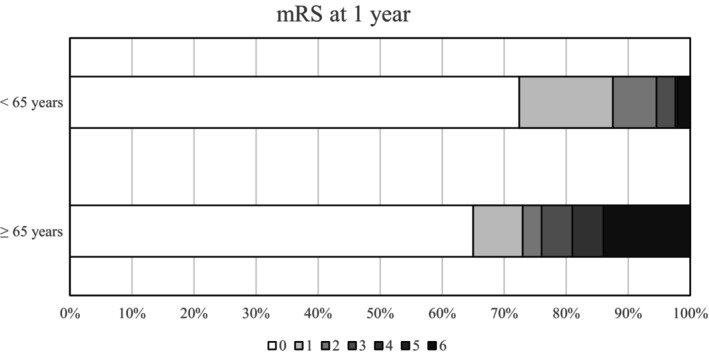
Comparison of the modified Rankin Score at 1 year in our cohort (114 cerebral venous thrombosis [CVT] patients aged ≥65 years) and the Lariboisière register (478 CVT patients aged <65 years). (Modified Rankin score 0: no symptoms; 1: no significant disability, despite symptoms; able to perform all usual duties and activities; 2: slight disability; unable to perform all previous activities but able to look after own affairs without assistance; 3: moderate disability; requires some help, but able to walk without assistance; 4: moderately severe disability; unable to walk without assistance and unable to attend to own bodily needs without assistance; 5: severe disability; bedridden, incontinent, and requires constant nursing care and attention; and 6: dead.)

The proportion of female patients was lower in our cohort than adults aged <65 years from the Lariboisière register (61% vs. 79%, *p* < 0.01; Table 3). Symptom onset was more frequently acute for patients in our cohort (46% vs. 19%, *p* < 0.01). Patients aged ≥65 years were significantly less prone to experience headaches, as an isolated symptom or not, than younger adults, but the older patients presented seizures and focal deficits more frequently (*p* < 0.01). Coma was also a more frequent feature among younger adults (22% vs. 10%, *p* < 0.01). Thrombosis location involved the lateral sinus or multiple sinuses more frequently for younger adults. The only brain injury that was significantly more frequent in our cohort compared with younger adults was arteriovenous dural fistulas (5% vs. 0.6%, *p* < 0.01). Concerning CVT risk factors, solid cancer and hemopathy, as well as local infection (intracranial, face, or the ENT area) were significantly more frequent in patients aged ≥65 years than in younger adults (14% vs. 2% for solid cancer, 7% vs. 2% for hemopathy, and 8% vs. 3% for local infection, *p* < 0.01). There was no difference in the proportion of acquired and nonacquired thrombophilia between groups. The death rate in the acute phase was significantly lower for younger adults (1.7% vs. 8%, *p* < 0.01). We also found a significant difference when looking at the mRS at 1 year, with a poorer outcome for patients from our cohort (*p* < 0.01; Table 3, Figure 2).

**TABLE 3:** Comparison of CVT characteristics in our patients aged ≥65 years and younger adults (18–64 years) from the Lariboisière register.

Characteristic	≥65 years CVT cohort, *N* = 114, *n* (%)	18–64 years CVT Lariboisière register, *N* = 478, *n* (%)	*p*
Female	69/114 (61)	377/478 (79)	**<0.01**
Symptom onset			
Acute	51/110 (46)	92/478 (19)	**<0.01**
Subacute	46/110 (42)	355/478 (74)	
Chronic	13/110 (12)	31/478 (6)	
Clinical presentation			
Isolated headache	21/114 (18)	151/478 (32)	**<0.01**
Headache	57/114 (50)	460/478 (96)	**<0.01**
Isolated intracranial hypertension	8/111 (7)	106/478 (22)	**<0.01**
Seizure	46/114 (40)	131/478 (27)	**<0.01**
Focal deficit	58/113 (51)	180/478 (38)	**<0.01**
Papilledema	7/40 (18)	160/477 (34)	**0.04**
Coma [Glasgow score ≤ 8]	11/114 (10)	103/478 (22)	**<0.01**
Thrombosis location			
Lateral sinus	79/114 (69)	380/478 (79)	**0.02**
Superior sagittal sinus	49/114 (43)	227/478 (47)	NS
Cortical vein	32/114 (28)	153/478 (32)	NS
Deep/straight vein	16/114 (14)	75/478 (16)	NS
Cavernous sinus	4/114 (3)	5/478 (1)	NS
Multiple locations	52/114 (46)	273/478 (57)	**0.03**
Brain injury			
Venous infarction	28/113 (25)	86/478 (18)	NS
Cerebral hemorrhage	34/113 (30)	119/478 (25)	NS
Subarachnoid hemorrhage	18/113 (16)	62/359 (17)	NS
Dural arteriovenous fistula	6/114 (5)	3/478 (0.6)	**<0.01**
Associated conditions			
Congenital thrombophilia	9/72 (13)	67/386 (17)	NS
Hemopathy	8/109 (7)	9/478 (2)	**<0.01**
Cancer [active]	15/109 (14)	11/478 (2)	**<0.01**
Local infection [face or intracranial]	9/109 (8)	12/478 (3)	**<0.01**
Nonlocal infection	5/109 (5)	10/478 (2)	NS
Antiphospholipid syndrome	5/71 (7)	14/478 (3)	NS
Anticoagulation	105/113 (93)	478/478 (100)	**<0.01**
Antiepileptic therapy	42/114 (37)	145/478 (30)	NS
Thrombolysis/thrombectomy	2/114 (2)	12/478 (2.5)	NS
Craniectomy	1/114 (1)	16/478 (3.3)	NS
Death in the acute phase	9/113 (8)	8/478 (1.7)	**<0.01**
mRS at 1 year			
0	41/63 (65)	339/469 (72)	**<0.01**
1	5/63 (8)	72/469 (15)	
2	2 /63 (3)	34/469 (7)	
3	3/63 (5)	12/469 (3)	
4	3/63 (5)	0/469 (0)	
5	0/63 (0)	2/469 (0.4)	
6	9/63 (14)	9/496 (2)	

Abbreviations: CVT, cerebral venous thrombosis; mRS, modified Rankin Scale; NS, not significant.

## DISCUSSION

We describe in this study a large cohort of elderly patients with CVT. We reported an annual incidence of 5.9 per million inhabitants for patients aged ≥65 years in the Paris–Ile‐de‐France region according to our cohort, which is close to the estimation in Ile‐de‐France (7.1 per million) and in France overall (8.5 per million) calculated from the national discharge database PMSI; this suggests we limited missing cases and selection bias [3, 4]. However, because some Ile‐de‐France hospitals did not participate, our cohort is not fully representative of the population of this region. In addition, because some patients may have been hospitalized in a geriatric department (especially beyond 75 years old), this bias was certainly not completely avoided. Furthermore, the incidence calculated from the PMSI certainly included some cases of fortuitously discovered CVT, and therefore could induce an overestimation of the incidence compared to the one calculated from our cohort.

Regarding differences between age subgroups, our study highlights some specificities concerning the clinical presentation of CVT among the older segment of the population, as they tend to have fewer headaches and signs of intracranial hypertension. By comparing subgroups of increasing age (65–74 years and ≥75 years) in our cohort, we found the probability of experiencing headaches with CVT decreases with age (as previously reported [6, 7]), whereas clinical signs of parenchymal damage (focal deficit) and cerebral/subarachnoid hemorrhage were more frequent. A plausible explanation would be that advancing age and comorbidities tend to reduce the volume of brain tissue, and thus the risk of intracranial hypertension in the case of sinus thrombosis. Regarding prognosis, our study highlights that mortality increases with age, as well as the probability of sequelae and reduction of functional independence. Among prognosis factors, data from initial evaluations appear to be crucial, such as the presence of a coma, the deep/straight vein location of CVT, and the presence of a venous infarction and/or hemorrhage. Of note, we also found 5% of our elderly cohort experienced CVT‐associated dural arteriovenous fistulas, consistent with a previous study that reported highly variable age of occurrence [9].

Regarding sex differences, we found a lower proportion of women with CVT in our cohort than among younger adults, but still constituting a slight majority (61%) [3, 4]. The more equilibrated sex ratio among older patients can be explained by the absence of sex hormone influence in postmenopausal women (including lack of oral contraceptive use, pregnancy, and puerperium); only 1.5% of our female CVT patients had HRT. However, even among the oldest part of the population, CVT remains a pathology that predominantly concerns women.

Concerning CVT‐associated risk factors and etiologies, despite possible different workup between younger and older patients, we found significantly more underlying cancer (14%), hemopathy (7%), and local infections (8%; of the ENT area or intracranial) in our cohort than for younger adults. Cancer and hemopathies have already been reported as more common in older patients [6, 7]. In the general population, systematic screening for cancer during CVT diagnosis is not recommended according to the European Stroke Organization guidelines, because it is found in only 5% of cases [10]. However, our study suggests that a thorough investigation for an underlying neoplasm may be more useful among patients aged >65 years. However, searching for cancer can also have negative effects (costs, incidental findings with potential harm for patients because of investigations, psychological effects). Because the study did not specifically address this topic, additional studies are needed before reaching conclusions. We can also add that *JAK2* mutations, when searched for, was found in a nonnegligible number of patients in our group, sometimes without any thrombocytosis or polyglobulia. A previous study reported *JAK2* mutation prevalence of 5.6% among patients with newly diagnosed CVT [11, 12]. Surprisingly, there was no significant difference in nonacquired inherited thrombophilia in older patients compared to younger adults (13% vs. 17%, respectively).

The strengths of this study include the large sample size and the diagnostic confirmation by robust methods in all cases. There are, however, potential limitations. The retrospective nature of this study impacted the quality and the quantity of available data regarding clinical features, etiological workup, and evaluation of outcome at 3 months and 1 year. Because the evaluation of outcome relied only on follow‐up consultation, information about patients who died after the acute phase was frequently missing. Also, systematic information on previous disability was not reported. The number of patients was too small to use multivariate analysis; therefore, we could only perform a univariate analysis to try to highlight the characteristics associated with prognosis. This can create confusion bias.

CVT after the age of 65 years is a rare disease with a higher risk of functional impairment that frequently reveals a potentially serious underlying pathology such as cancer. Its early detection and a thorough etiological assessment are of paramount importance. Anticoagulation is the key treatment, together with the specific management of any underlying associated pathology. This study gives insight into CVT in the older segment of the population and compels us not to rule out the diagnosis even in the absence of headaches for older patients, to facilitate CT or magnetic resonance venous angiography when facing an elderly patient with neurological symptoms of undetermined origin, and to track down any underlying prothrombotic pathology.

## AUTHOR CONTRIBUTIONS

**Victor Garcia:** Investigation; writing – original draft; methodology. **Louise Bicart‐Sée:** Investigation; writing – original draft; supervision. **Isabelle Crassard:** Validation; investigation; writing – review and editing. **Nicolas Legris:** Investigation; conceptualization; methodology; supervision; writing – review and editing. **Mathieu Zuber:** Investigation; writing – review and editing. **Fernando Pico:** Investigation; writing – review and editing. **Céline Guidoux:** Investigation; writing – review and editing. **Michael Obadia:** Investigation; writing – review and editing. **Naouel Boulenoir:** Investigation. **Didier Smadja:** Investigation. **Mikael Mazighi:** Investigation. **Cecile Lavenu‐Bombled:** Investigation; writing – review and editing. **Elodie Baudry:** Investigation. **Bertrand Lapergue:** Investigation. **Guillaume Turc:** Investigation. **Philippe Tuppin:** Investigation; conceptualization; methodology; supervision; data curation. **Christian Denier:** Conceptualization; investigation; writing – original draft; validation; methodology; supervision; data curation.

## CONFLICT OF INTEREST STATEMENT

The authors declare no competing interests.

## Data Availability

Data are available upon reasonable request.
